# COVID-19 outbreak at a residential apartment building in Northern Ontario, Canada

**DOI:** 10.1017/S0950268824000256

**Published:** 2024-03-04

**Authors:** Dinna Lozano, Carolyn Dohoo, David Elfstrom, Kendra Carswell, Jennifer L. Guthrie

**Affiliations:** 1 North Bay Parry Sound District Health Unit, North Bay, ON, Canada; 2 Public Health Agency of Canada, Ottawa, ON, Canada; 3 Independent Consultant, Stratford, ON, Canada; 4 Department of Microbiology & Immunology, Western University, London, ON, Canada; 5 Public Health Ontario, Toronto, ON, Canada

**Keywords:** COVID-19, multi-unit residential building, outbreaks, SARS-CoV-2, transmission

## Abstract

In February 2021, a cluster of Beta variant (B.1.351) coronavirus disease 2019 (COVID-19) cases were identified in an apartment building located in Northern Ontario, Canada. Most cases had no known contact with each other. Objectives of this multi-component outbreak investigation were to better understand the social and environmental factors that facilitated the transmission of COVID-19 through this multi-unit residential building (MURB). A case–control study examined building-specific exposures and resident behaviours that may have increased the odds of being a case. A professional engineer assessed the building’s heating, ventilation, and air-conditioning (HVAC) systems. Whole-genome sequencing and an in-depth genomic analysis were performed. Forty-five outbreak-confirmed cases were identified. From the case–control study, being on the upper floors (OR: 10.4; 95% CI: 1.63–66.9) and within three adjacent vertical lines (OR: 28.3; 3.57–225) were both significantly associated with being a case of COVID-19, after adjusting for age. There were no significant differences in reported behaviours, use of shared spaces, or precautions taken between cases and controls. An assessment of the building’s ventilation found uncontrolled air leakage between apartment units. A single genomic cluster was identified, where most sequences were identical to one another. Findings from the multiple components of this investigation are suggestive of aerosol transmission between units.

## Introduction

Current evidence indicates the transmission of severe acute respiratory syndrome coronavirus 2 (SARS-CoV-2) varies across respiratory particle size and distances, with increased risk at shorter distances between the source and the receptor (e.g. face-to-face discussion) [1, 2]. Factors favourable to the transmission of SARS-CoV-2 across longer distances include inadequate ventilation, prolonged exposure, high viral load, particle size, and certain activities [1].

In addition to the potential for SARS-CoV-2 to transmit through close contact [1], transmission in multi-unit residential buildings (MURBs) may occur through aerosols or fomites in shared spaces, inadequate or faulty ventilation or plumbing systems, and unintentional airflow [3]. Despite the developing literature, outbreak investigations of coronavirus disease 2019 (COVID-19) in apartment/condominium settings have not been extensively documented in Canada.

On 28 January 2021, two confirmed cases of COVID-19 were reported to the North Bay Parry Sound District Health Unit (Health Unit), both residents of the same apartment building located within the Health Unit’s service area. Case investigations identified that the index case likely acquired infection during international travel, and these cases were known contacts of each other. Two more confirmed cases of COVID-19 who were residents of the same apartment building and had no known contact with earlier cases were identified between 4 and 6 February 2021. This prompted further investigation into this cluster and on-site testing. By 8 February 2021, 18 additional residents or visitors to the building tested positive for COVID-19 and an outbreak was declared by the local medical officer of health. On 13 February 2021, whole-genome sequencing identified that the lineage of COVID-19 was the Beta variant (B.1.351). This variant was detected in Ontario, Canada, in early 2021, and at the time, it was presumed to be more transmissible than the original Wuhan-Hu-1 strain [4]. Early cases reported in connection with the outbreak resided in units located vertically above each other and did not report contact with each other, which prompted a comprehensive, multidisciplinary investigation.

The objectives of this outbreak investigation were to better understand the social and environmental factors that facilitated the transmission of COVID-19 through this MURB. Findings from this comprehensive investigation that included a case–control study, environmental assessment, and laboratory investigation may contribute to a better understanding of COVID-19 transmission and outbreaks within mid-rise MURBs in Canada.

## Methods

### Setting

The seven-storey building in this outbreak was built in the 1970s, with 98 units (bachelor, one bedroom, two bedrooms) and approximately 139 residents at the time of the outbreak. Shared common areas included the garbage room, mail room, laundry room, main entrance, lobby, elevator, stairwells, and hallways.

### Case finding

Cases were defined according to the provincial case definition [5] at the time of the outbreak, and they were visitors, residents, or staff working in the apartment building, with an accurate episode date between 20 January 2021 and 23 March 2021. In all analyses, the accurate episode date was defined by the earliest date of symptom onset date, specimen collection date, or reported date.

Two on-site testing clinics (7 February 2021 and 16 February 2021) and one drive-through testing clinic (7 March 2021) were offered by the local COVID-19 Assessment Centre for residents of the building.

### Descriptive epidemiology

Data from case investigation and contact tracing activities were extracted from the Provincial Case and Contact Management Solution (CCM) database. Descriptive epidemiology analyses included counts, proportions, and an epidemic curve. Attack rates among residents of the building were calculated [6].

### Unit-level analyses

Data from 91 of 98 units in the building (one unit was confirmed vacant and six additional units had no laboratory testing completed) were analysed using logistic regression to determine the association between a unit containing at least one case of COVID-19 and the unit location within the building.

### Case–control study

A case–control study was conducted to identify potential exposures associated with COVID-19 infection and to inform transmission dynamics of SARS-CoV-2 in the outbreak setting. The study was reviewed and approved by the Health Unit’s Research Ethics Review Committee.

**Figure 1.**
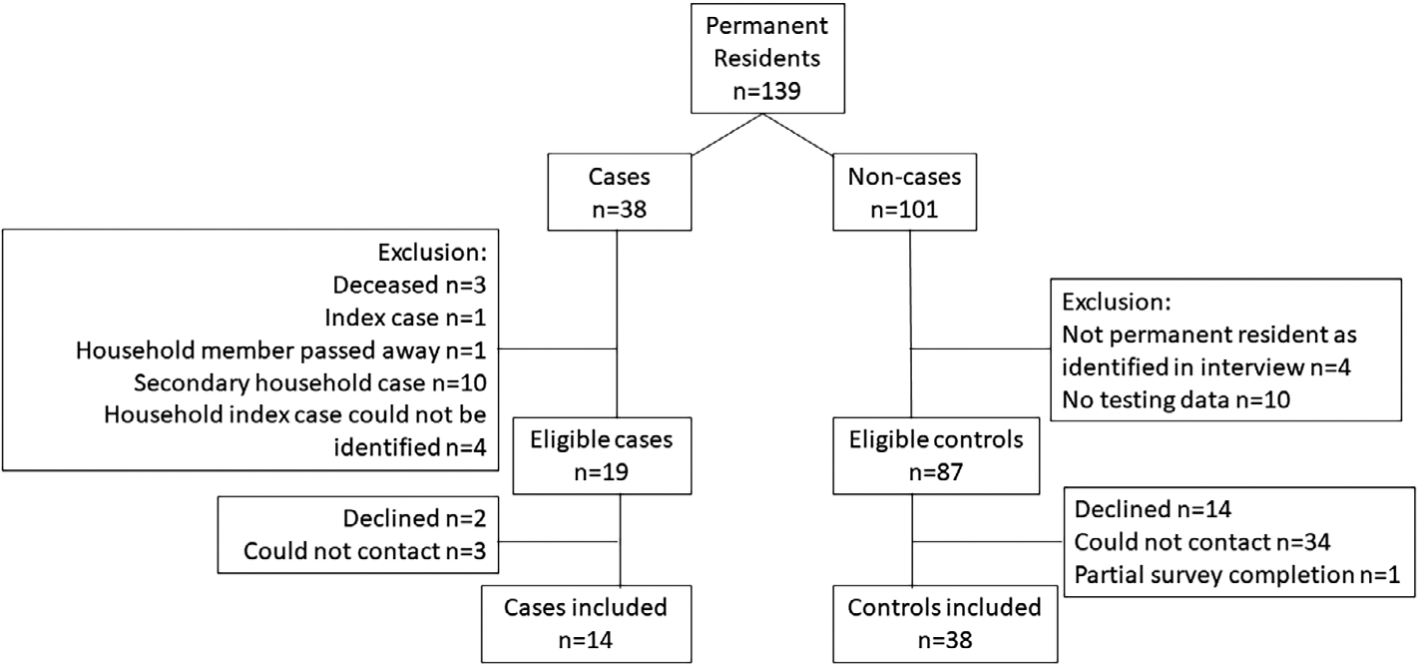
Case and control eligibility and attrition

The population for the study included all permanent residents of the building between 13 January and 7 March 2021. Eligible cases included those with evidence of being the index case within a unit, when a unit had more than one confirmed case of COVID-19 associated with the outbreak. Eligible controls were permanent residents of the apartment building during the time period of interest, with a negative laboratory result for COVID-19. Full inclusion and exclusion criteria for participation in this study are presented in Table 1 and Figure 1. Detailed methods are presented in the Supplementary Material available on the Cambridge Core website.

**Table 1.** Case and control inclusion and exclusion criteria

Inclusion criteria	Exclusion criteria
*Cases*	
Confirmed cases of COVID-19 who were permanent residents of the apartment building between 13 January and 7 March 2021 with: An accurate episode date Evidence of being the index case within a unit, when a unit had more than one confirmed case of COVID-19 associated with the outbreak	Confirmed cases of COVID-19 associated with the outbreak who were: Temporary residents, visitors, or non-resident staff Deceased Cases occurring within a unit where the index case for the unit had already been identified and included Cases in a unit with more than one case where an index case could not be identified (e.g. two asymptomatic cases with testing results from the same day) The outbreak index case
*Controls*	
Permanent residents of the apartment building between 13 January and 7 March 2021 with a negative laboratory result for COVID-19	Temporary residents, visitors, and non-resident staff of the apartment building between 13 January and 7 March 2021

Between 16 and 26 March 2021, a questionnaire was administered to participating residents to gather data on demographics, medical and behavioural risk factors, building exposures, and apartment unit features within the 2 weeks prior to the accurate episode date for cases and a matched time period for non-cases.

Descriptive statistics and univariable logistic regression were used to quantify the associations between exposure/risk factor variables and COVID-19 status. Due to the small number in the case–control study, all variables included were dichotomized. For those variables with a crude association (*p* < 0.2), a multivariable logistic regression model was built using non-automated, backward selection principles. Variables were removed from the full model based on significance, and confounding was considered if the direction and magnitude of the remaining coefficients changed (>30% change). Within the logistic regression analyses, records with missing or ‘don’t know’ values among each exposure were dropped by list-wise deletion. Data cleaning, manipulation, and analysis were conducted in Stata/IC 15.1.

### Laboratory investigation

Polymerase chain reaction diagnostic testing was performed on clinical specimens using the Applied Biosystems TaqPath™ COVID-19 Assay or BioFire® FilmArray Respiratory Panel 2.1 Assay at a hospital laboratory. Specimens were forwarded to the Public Health Ontario Laboratory (PHOL) where they were tested for the gene mutations (501Y, K147N, and E484K) associated with variants of concern, if there was adequate viral load (cycle threshold (Ct) value ≤35) [7]. Whole-genome sequencing and an in-depth genomic analysis were also performed at PHOL to identify genomic clusters and the possibility of multiple introductions, where specimens were available with sufficient volume remaining and of adequate viral quantity (Ct ≤30). As part of quality control, we determined the genome completeness of each sequence compared to the Wuhan-Hu-1 reference genome as a percentage and calculated the overall mean and standard deviation (SD). See the Supplementary Material available on the Cambridge Core website for whole-genome sequencing and bioinformatic methods.

### Environmental investigation

A professional engineer contracted by the Health Unit performed an on-site assessment (27 February 2021) of the building’s heating, ventilation, and air-conditioning (HVAC) systems and assessed how air moved through the building. Visual observations and measurements of airflows and pressure differences were gathered on building and unit-level heating and ventilation components, including the makeup air system, exhaust systems, portable air cleaners, and air leakage pathways.

## Interventions

### Case and contact management

Cases and contacts of COVID-19 were managed as per provincial guidelines that outlined the duration of isolation, traceback periods for identifying contacts, and education related to infection prevention and control [8]. On 20 March 2021, the Health Unit held an on-site COVID-19 vaccination clinic.

### Community interventions

In Ontario, a province-wide stay-at-home order was in place at the time of the outbreak, where public and social gatherings were restricted, mask or face coverings were mandated within open indoor businesses and organizations, and non-essential retail stores had restricted hours of operation [9]. The Health Unit area remained under this order until 8 March 2021 to prevent the variant from this outbreak spreading further into the community [9, 10].

### Building interventions

After the outbreak was identified, guidance and recommendations from the American Society of Heating, Refrigerating and Air-Conditioning Engineers [11] were provided to residents, on-site staff, and building management. On 16 February 2021, 17 portable air cleaners with 2,200 m^3^/h high-efficiency particulate air (HEPA)-filtered airflow each were placed at both ends of the corridor on every floor and in three common areas. The air cleaners provided substantial equivalent air changes (15–49 air changes per hour), which were above the target Non-Infectious Air Delivery Rates for reducing exposure to airborne respiratory diseases recently proposed by the Lancet COVID Commission Task Force [12]. Directions and an audit on proper cleaning and disinfection practices were provided by the Health Unit to building staff.

## Results

### Descriptive epidemiology

In all, 45 outbreak-confirmed cases were identified. Most cases were permanent residents (*n* = 38) of the building, with seven temporary residents and visitors. Permanent and temporary resident cases resided in 26 separate apartment units. A similar number of cases were male versus female, and the median age of cases was 58 years (range: 5–84) (Table 2). Thirteen per cent of cases were asymptomatic at the time of testing. Five cases (11.1%) were hospitalized, and three cases (6.7%) died, where COVID-19 was determined to be the underlying cause of death.

**Table 2.** Number and percentage of outbreak-confirmed COVID-19 cases by demographic variables (*n* = 45)

Variable	*n* (%)
*Sex*	
Female	22 (48.9)
Male	23 (51.1)
*Age group (years)*	
0–39 years	10 (22.2)
40–49 years	8 (17.8)
50–59 years	6 (13.3)
60–69 years	9 (20.0)
70–79 years	7 (15.6)
80 years or older	5 (11.1)
*Apartment building floor of residence*	
Floors 1–4	16 (35.6)
Floors 5–7	22 (48.9)
Temporary residents/visitors	7 (15.6)

**Figure 2.**
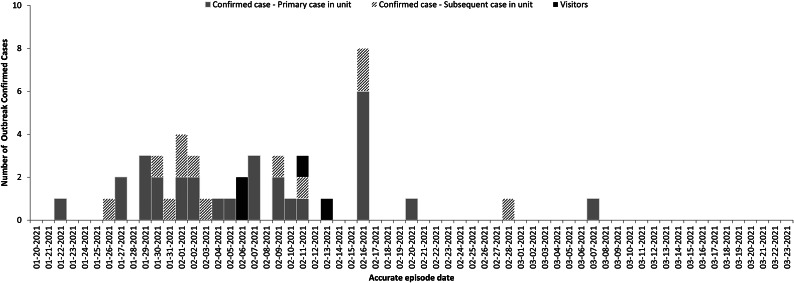
Number of outbreak confirmed COVID-19 cases by accurate episode date, 22 January 2021 – 7 March 2021 (n=45)

A small peak in cases was observed on 1 February 2021, with a larger peak on 16 February 2021 (Figure 2). Initial mapping of cases according to their apartment unit of residence showed clustering in units that were vertically aligned in three adjacent columns, located proximately above the unit where the index case resided, herein referred to as the vertical lines. The overall attack rate for resident cases was 28.9% (95% CI: 20.7, 39.2). The primary attack rate was 20.4% (95% CI: 13.7, 29.3). The household secondary attack rate was 91.7% (95% CI: 45.8%, 164.0%).

### Unit-level analyses

Results from the unit-level logistic regression demonstrated that the location within the building, both location on upper three floors (OR: 4.55; 95% CI: 1.34–15.4) and within the vertical lines in the building (OR: 18.9; 95% CI: 5.10–69.8), was significantly associated with increased odds of having a case of COVID-19 (Table 3). There was no statistically significant interaction between floor and vertical lines in the final model.

**Table 3.** Unit-level logistic regression model of the association between location in building and having a case of COVID-19 (*n* = 91)

Variable	OR^a^	95% CI^b^	*p*-value
Floor of residence (upper)^c^	4.55	1.34–15.4	0.02
In vertical lines^d^	18.9	5.10–69.8	<0.01
Constant	0.08	0.03–0.23	<0.01

a Odds ratio (OR).

b Confidence interval (CI).

c Lower floors of residence: 1–4; upper floors: 5–7.

d Vertical lines defined as units that were vertically aligned in three adjacent columns, located proximately above the unit where the index case resided.

### Case–control study

Fourteen cases and 38 controls were recruited into the study (Figure 1). Cases were younger in age than controls and were more likely to reside on upper floors or in the vertical lines in the building (Table 4). Most cases and controls (>70%) spent on average >22 h per day in the building (Table 4). There were no significant differences between cases and controls in reported personal protective measures such as hand hygiene or in the use of shared spaces (Table 4). No one reported close contact interactions (less than two metres for at least 15 min) [8] with individuals outside of the household (Table 4), and any reported interactions with others in the building were transient, such as brief hallway chats (Supplementary Table S2).

**Table 4.** Frequency and univariable logistic regression associations from the case–control study (*n* = 52)

Variable	*n* (%) cases	*n* (%) controls	OR (95% CI)^a^	*p*-value
Age ≥ 50 years	7	30	0.97	0.05
	(50.0)	(79.0)	(0.95–1.00)	
Sex (female)	7	26	2.17	0.23
	(50.0)	(68.4)	(0.62–7.57)	
Reported race category, white (European descent)	11	37	6.73	0.13
	(78.6)	(97.4)	(0.56–81.4)	
Income (≥$30,000)	7	4	2.05	0.30
	(50.0)	(10.5)	(0.53–7.87)	
Reported any medical risk factor	8	23	0.87	0.83
	(57.1)	(60.5)	(0.25–3.01)	
Resided on upper floors^b^	10	10	4.81	0.02
	(71.4)	(26.3)	(1.26–18.4)	
Resided in vertical lines^c^	8	5	8.80	<0.01
	(57.1)	(13.2)	(2.14–36.3)	
Reported hand hygiene	14	33	N/A	N/A
	(100)	(86.8)		
Reported physical distancing	13	34	N/A	N/A
	(92.9)	(89.5)		
Reported mask use	14	35	N/A	N/A
	(100)	(92.1)		
Reported close contact interactions	0	0	N/A	N/A
	(0.00)	(0.00)		
Reported stair use	2	9	0.59	0.53
	(14.3)	(23.9)	(0.11–3.15)	
Reported using the mailroom	9	27	0.73	0.64
	(64.3)	(71.1)	(0.20–2.69)	
Reported using the garbage room	9	20	1.62	0.46
	(64.3)	(52.6)	(0.46–5.74)	
Reporting using the lobby	13	31	2.94	0.34
	(92.9)	(81.6)	(0.33–26.3)	
Reported using the laundry room	8	16	1.75	0.38
	(57.1)	(42.1)	(0.51–6.06)	
Reported any elevator use	13	27	5.30	0.13
	(92.9)	(71.1)	(0.62–45.5)	
≥22 h per day spent in the building	10	29	0.98	0.49
	(71.4)	(76.3)	(0.92–1.04)	

Odds ratios and p-values for odds ratios were not calculated for variables with low variation (zero cells) and are represented here by values of ‘N/A’. They were excluded from the multivariable logistic regression analysis.

a Odds ratio (OR), confidence interval (CI)

b Lower floors of residence: 1–4; upper floors: 5–7

c Vertical lines defined as units that were vertically aligned in three adjacent columns, located proximately above the unit where the index case resided.

Findings from the multivariable logistic regression analysis demonstrate that location within the apartment building, both the location of cases by floor (living on floors 5–7; OR: 10.4; 95% CI: 1.63–67.9) and within the vertical lines (OR: 28.3; 95% CI: 3.57–225), was associated with increased odds of being a case, after controlling for age (Table 5). There was no statistically significant interaction between floor and vertical lines in the final model.

**Table 5.** Multivariable logistic regression of the risk factors associated with being a case of COVID-19 (*n* = 52)

Variable	OR^a^	95% CI^b^	*p*-value
Age (≥50 years)	0.95	0.92–0.99	0.01
Floor of residence (upper)^c^	10.4	1.63–67.9	0.01
In vertical lines^d^	28.3	3.57–225	<0.01
Constant	0.15	0.03–0.87	0.03

a Odds ratio (OR).

b Confidence interval (CI).

c Lower floors of residence: 1–4; upper floors: 5–7.

d Vertical lines defined as units that were vertically aligned in three adjacent columns, located proximately above the unit where the index case resided.

### Laboratory investigation

**Figure 3.**
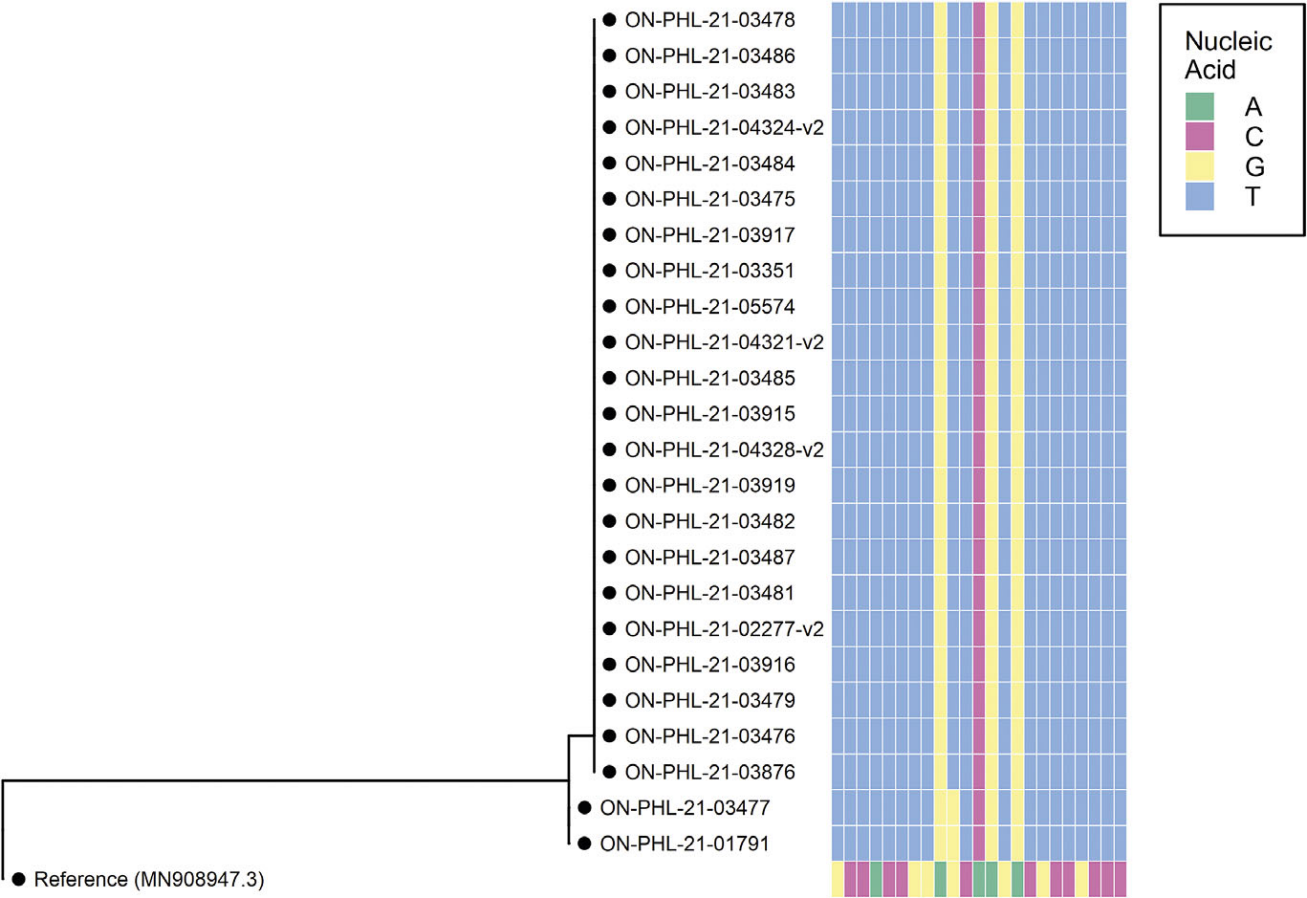
A maximum-likelihood phylogenetic tree depicting the evolutionary relationships of SARS-CoV-2 genomes obtained from outbreak-related resident case specimens, built using IQ-TREE based on single nucleotide polymorphisms (SNPs) from the Wuhan Hu-1 reference (Genbank Accession MN908947.3); SNP profile is displayed as nucleic acids

One hundred and twenty-nine of the 139 residents were tested for COVID-19. Most cases (62.2%) had the Beta variant (lineage B.1.351) detected through whole-genome sequencing (Table 6). From an in-depth genomic analysis of 24 cases that generated high-quality sequence data with a mean genome completeness of 98.5% (SD ± 0.7), a single genomic cluster was identified. The majority (22/24; 91.7%) of sequences were identical to one another and differentiated from two sequences (including the index case) that were separated by a single-nucleotide polymorphism (SNP) (Figure 3).

**Table 6.** Number and percentage of outbreak-confirmed COVID-19 cases by variant of concern result (*n* = 45)

Variant of concern	*n* (%)
Screened positive for mutation N501Y	8 (17.8)
Beta variant (lineage B.1.351)	28 (62.2)
Unable to test	9 (20.0)

### Environmental investigation

Ventilation to the apartment building was a standard design, consisting of a makeup air unit on the roof providing outdoor air to each corridor. Exhaust fans located on the roof provided centralized kitchen and washroom exhaust via shafts. In this design, heated outdoor air is sent to corridors at a positive pressure relative to the units, which are at lower pressure due to the exhaust. A gap under each unit door facilitates the air transfer into the units.

The assessment of the building at the time of the outbreak revealed approximately four times more air was continually exhausted relative to outdoor air supplied by the makeup air unit. There were common areas of the building with no mechanical ventilation, including the mail room and laundry room. Makeup air delivered to the corridors was greatest on the top floor and the least on the ground floor, and upper-level units had greater exhaust rates than lower units.

Evidence for uncontrolled airflow was found in one unit on the top floor and within the vertical lines of interest, where air was rapidly flowing into the unit through both kitchen (2.3 L/s) and washroom unsealed plumbing penetrations under the sinks, pulled in by the strong exhaust draw. A faint sour smell was detected under the sink in the same unit, which may have indicated contamination from a cracked or corroded soil stack or drain, or from a roof drain open to the sanitary sewer.

## Discussion

This outbreak report describes a comprehensive investigation of the rapid transmission of COVID-19 within a MURB where 28.9% of residents were infected. This was during a time when community transmission of COVID-19 was low, and vaccines were not available to the local general population. As the Beta variant was not identified in the community prior to or during the outbreak and a single genomic cluster was identified, it is unlikely that the transmission of this strain occurred by multiple introductions into the building by separate individuals. The location of residents within the building, particularly within the vertical lines and residences on upper floors, was significantly associated with COVID-19 infection in this outbreak. Coupled with findings from engineer inspections of the building and the case–control study, the results are suggestive of possible aerosol transmission between units.

Unintentional air movement may have contributed to the spread of the virus in this outbreak. An air leakage of the leakage class massive (detectable by hand, 0.3 m (1 ft.) from the leak) [13] was found in one unit around unsealed plumbing penetrations. The building was undersupplied for outdoor air relative to the amount of air exhausted from the building. It is surmised that the high exhaust flow drew outdoor air into the building through the building envelope (e.g. cracks and open windows). Air could then travel between units via cracks such as unsealed plumbing penetrations, wiring raceways, HVAC duct risers, plumbing risers, and stack penetrations, until it exited out the exhaust of the building [13]. In doing so, the air could carry contaminants, including infectious aerosols, from one location to another [14]. An investigation of COVID-19 transmission in a Taiwanese quarantine facility found evidence of aerosol transmission between inadequately ventilated units through structural defects or gaps around building components, verified by visual inspection and the use of a simplified tracer gas experiment [15]. Transmission related to contaminated air transported through plumbing components has been reported in previous investigations [16–21]. In this building, sour smells were observed in one unit under the sink where air was rapidly flowing into the unit through unsealed plumbing penetrations. Observed sour smells could have been due to a cracked sewer drain or vent stacks; however, this was not assessed. Sewer gas or contaminated air from dried-out traps was a possible area of infiltration; however, this was unlikely in an occupied building where most units had a single washroom. There were multiple possible air leakage pathways in this apartment building that could have led to the transmission of COVID-19 between units.

Units located both on the upper three floors and within the vertical lines proximately above the index case’s unit were significantly associated with increased odds of having a case of COVID-19. The airflow in this apartment building, due to the difference between outdoor and indoor temperatures along with any wind-induced pressure differentials, may have driven air to be distributed vertically and sideways between apartment units via pressure differences. In buildings in cold climates such as Canada during the heating season, a stack effect occurs where cold air leaks in through the lower portion of the building, is heated, and then travels to the upper portion where it leaks outwards [13]. The stack effect is stronger the higher the building structure, and the greater the differential between indoor and outdoor air temperatures. This outbreak occurred during the winter months in Northern Ontario (median average daily outdoor temperature: −13.6°C; estimated indoor temperature: 21–24°C) [22, 23]. COVID-19 transmission along vertical lines of apartment buildings has been previously described in the literature. A version of the stack effect was thought to have contributed to the spread among residents in vertically oriented apartment units within COVID-19 outbreaks in Asia [16–21]. A modelling study in MURBs in cold climates showed airborne contaminants originating in lower-level units can accumulate in upper-level units due to a combination of air leakage and the stack effect [14]. This vertical pattern of transmission provides evidence for aerosol transmission over longer distances.

There is a greater risk of infection with COVID-19 at shorter distances from an infectious source, especially without protective measures in place [1, 2]. In the case–control study results, we found no significant differences between cases and controls in reported personal protective measures, including hand hygiene, masking, physical distancing, and no close contact interactions, were reported. No mechanical ventilation was identified in the mail room or laundry room, which could be areas of the building with increased risk for transmission of COVID-19 due to poor ventilation [24–26]. However, the reported use of shared spaces, including the mail and laundry rooms, did not differ significantly between cases and controls in our investigation. Case–control study results, interpreted in the context of the full outbreak investigation, present low evidence for transmission through short-range transmission (e.g. face-to-face discussion, use of shared spaces) outside of households. However, because of sample size limitations, these avenues cannot be definitively ruled out.

A limitation of the case–control study was the small sample size; there was only sufficient power to detect large effect sizes. Selection bias was a possibility as this was a case–control study where participation among eligible cases and controls was voluntary, and systematic differences may have existed between those who were able to be contacted and agreed to participate and those who did not. Misclassification bias was possible among controls if individuals were only tested during their incubation period or infrequently during the outbreak. However, repeat testing was available during the outbreak and 60.5% of controls were tested three or more times, 26.3% were tested two times, and only 13.2% were tested one time. Respondents may have been impacted by recall bias as they were asked about their general health behaviours up to 2 months prior to the interview. To reduce this bias, calendars and memory aids were suggested for use. At the time of interview, there were considerable public health restrictions and media attention related to the outbreak, which may have influenced respondents’ self-reported behaviours towards compliance with public health recommendations. To reduce this, respondents were informed that there would be no consequences for any individual responses provided or participation in the study. Portable air cleaners were put in place on 16 February 2021; only three cases occurred after this date. However, we were unable to assess the impact of each public health intervention as multiple interventions were generally applied within the span of the 14-day maximum incubation period. Certain aspects of an environmental engineering assessment, including a tracer gas study, could not be performed due to feasibility or missing information such as mechanical drawings of the HVAC system. A limitation of the in-depth genomic analysis was that only approximately half of the specimens were of high enough viral quantity or available for inclusion in the analysis.

A strength of this overall investigation was that it used a multifactorial approach including a case–control study design to assess potential exposures (reported behaviours, use of shared spaces, or precautions taken), with supporting evidence from environmental and laboratory investigations to inform an overall understanding of transmission events. There are limited publications that describe COVID-19 outbreaks in MURBs, particularly in North America, and few investigations that use a multifactorial approach. The findings from the multiple components of this investigation suggest that inter-unit aerosol transmission over longer distances may have contributed to the rapid progression of a COVID-19 outbreak in this older Canadian MURB. This may be an under-considered risk for these types of buildings, constructed with unsealed gaps and cracks between units, creating a greater chance of unplanned airflow due to pressure differentials. As recommended in the *American Society of Heating, Refrigerating and Air Conditioning (ASHRAE) Standard 241 Control of Infectious Aerosols* [27], optimizing building ventilation system performance and sealing penetrations, gaps, or cracks between units could improve indoor air quality and prevent contaminated air from being shared between units. Future multidisciplinary investigations, as was done in this study, may help to confirm the modes of transmission of respiratory infections in MURBs to inform public health interventions and building codes.

## Supporting information

Lozano et al. supplementary material Lozano et al. supplementary material

## Data Availability

The data from this paper are not publicly available due to privacy concerns and legislative requirements.

## Supplementary material

For supplementary material accompanying this paper visit http://doi.org/10.1017/S0950268824000256. click here to view supplementary material
