# Supplementary material for: COVID-19 outbreak at a residential apartment building in Northern Ontario, Canada
Source: Epidemiol Infect. 2024 Mar 4;152:e53. doi: 10.1017/S0950268824000256 (PMC11022249; doi:10.1017/S0950268824000256)
Supplement: Lozano et al. supplementary material [file S0950268824000256sup001.pdf]

Epidemiology and Infection

COVID-19 outbreak at a residential apartment building in Northern Ontario, Canada

Dinna Lozano, Carolyn Dohoo, David Elfstrom, Kendra Carswell, Jennifer L. Guthrie

Supplementary Material

Detailed methods and additional results for the case-control study and in-depth genomic analysis.

Methods: Case-control study

Participant selection & notification

A list of residents provided by building management, combined with a list of all residents tested for COVID-19 at on-site testing clinics, was used to identify non-case residents of the building for control selection. A case-control study information package was delivered to the units of all eligible cases and controls to provide notification of the study and to expect contact for this purpose.

Data sources

1) Ontario Case and Contact Management database (CCM)

2) Ontario Laboratories Information System (OLIS)

3) Apartment resident list (from building management)

4) Study questionnaire

Data collection

The full questionnaire contained questions specific to the case-control study as well as questions to inform other aspects of the outbreak investigation and environmental inspection. For cases, the questionnaire referred to general individual behaviour in the two weeks prior to either symptom onset or positive test date. To account for the possibility of changes in behaviour among the control group, depending on knowledge of the outbreak, the two-week period for which controls were referred was randomly matched to the case date ranges. Control questionnaires contained additional questions related to demographics, medical risk factors, and vaccination status, to match what was available for cases in the CCM database.

Control interviews were conducted by an independent research company and case interviews were conducted by public health staff supporting the outbreak investigation. Data from the questionnaire was collected predominantly through phone interview, with an online and paper version available upon request. For each eligible case and control, three phone call attempts were made, one in each of the morning, afternoon, and evening.

Data from CCM and OLIS were extracted for cases and controls and linked to the questionnaire responses.

Analysis

Descriptive statistics (counts, proportions, means, medians, ranges) and comparisons (Fisher exact test for categorical variables and two-tailed t-test for continuous variables) between the case and control groups were conducted for variables collected (significance, $p \leq 0.05$).

Data analysis of questions specific to the case-control study are presented in the Results section and Supplementary Table 1. In situations where multiple questions were measuring the same behaviour or

exposure, decisions were made about the most appropriate variable to include in the data analysis based on the data quality and reliability.

Considerations for handling multiple controls that are living in the same unit were as follows. During descriptive and univariable analysis, individual-level factors were analysed using all cases and controls but for unit-level factors only one control per unit was included. For multivariable model building, all observations were included in the model building process and then the same process was repeated after dropping an observation from units that had two cases or controls, to assess the impact of clustering within unit on the multivariable analysis results. As expected with a maximum of two cases per cluster, the impact was limited, and results are not presented.

Methods: Whole genome sequencing and bioinformatics analysis

RNA was isolated from SARS-CoV-2 positive specimens, and complementary DNA (cDNA) was synthesized using established protocols. To generate ARTIC V3 amplicons, we followed standard methods, and subsequently, genomic libraries were prepared using the Illumina's Nextera XT DNA Library Preparation Kit. Paired-end sequencing of genomes was conducted on an Illumina MiSeq or NextSeq 550 instrument, producing reads of 2 × 150 base pairs.

For bioinformatic analysis, we utilized a modified version of the COVID-19 Genomics UK (COG-UK) pipeline, which involved reference-based assembly and variant calling using the GenBank accession MN908947 (https://github.com/oicr-gsi/ncov2019-artic-nf). To ensure sequencing quality, we performed quality control using ncov-tools (http://github.com/jts/ncov-tools). We aligned the resulting consensus FASTA sequences with MAFFT (v7.471) [1] and masked the 5′ and 3′ ends of the sequences corresponding to positions 1–54 and 29837–29903 in the reference, respectively. Genome completeness was calculated for each sequence as a percentage of the alignment to the reference genome. Samples that generated high quality data are those with a genome completeness of ≥90%.

A maximum-likelihood phylogeny was constructed using IQ-TREE (v2.0.3) [2] and annotated using the ggtree package [3] in R (v4.3.0). We used the Phylogenetic Assignment of Named Global Outbreak Lineages (PANGOLIN) software to predict SARS-CoV-2 lineage (pangolin 3.1.16, pangoLEARN 2021-10-18) [4]. Consensus sequences are available in GISAID (https://www.gisaid.org/), Supplementary Table 1.

Supplementary Table S1 GISAID and NCBI's Sequence Read Archive accession numbers for whole genome sequenced SARS-CoV-2 samples.

Sample Id	GISAID[a] Accession	SRA[b] Accession
ON-PHL-21-03487	EPI_ISL_1230397	SRR25429297
ON-PHL-21-05574	EPI_ISL_1368422	SRR25429296
ON-PHL-21-03876	EPI_ISL_1334611	SRR25429285
ON-PHL-21-03485	EPI_ISL_1229994	SRR25429280
ON-PHL-21-03915	EPI_ISL_1501411	SRR25429279
ON-PHL-21-03478	EPI_ISL_1230012	SRR25429278
ON-PHL-21-03477	EPI_ISL_1501359	SRR25429277
ON-PHL-21-03351	EPI_ISL_1501347	SRR25429276
ON-PHL-21-03919	EPI_ISL_1501413	SRR25429275
ON-PHL-21-03486	EPI_ISL_1230396	SRR25429274
ON-PHL-21-03484	EPI_ISL_1230395	SRR25429295
ON-PHL-21-02277-v2	EPI_ISL_1501405	SRR25429294
ON-PHL-21-01791	EPI_ISL_1183114	SRR25429293
ON-PHL-21-03479	EPI_ISL_1501360	SRR25429292
ON-PHL-21-03482	EPI_ISL_1501361	SRR25429291

ON-PHL-21-03483	EPI_ISL_1501362	SRR25429290
ON-PHL-21-03481	EPI_ISL_1230394	SRR25429289
ON-PHL-21-03917	EPI_ISL_1501412	SRR25429288
ON-PHL-21-03916	EPI_ISL_1335004	SRR25429287
ON-PHL-21-04328-v2	EPI_ISL_1501508	SRR25429286
ON-PHL-21-04324-v2	EPI_ISL_1501506	SRR25429284
ON-PHL-21-04321-v2	EPI_ISL_1501505	SRR25429283
ON-PHL-21-03476	EPI_ISL_1230393	SRR25429282
ON-PHL-21-03475	EPI_ISL_1501358	SRR25429281

[a] Global Initiative on Sharing All Influenza Data

[b] Sequence Read Archive

Results: Case-control study

Few cases and controls reported having visitors, entering other residents' apartments, or receiving personal services (e.g., food delivery, housekeeping) in their units.

Supplementary Table S2 Frequency and univariable associations from the case-control study for additional social interaction variables (n=52)

Variable	n (%) cases	n (%) controls	OR (95% CI)[a]	p-value
Reported brief encounters with other building residents	7 (50.0)	20 (52.6)	0.90 (0.26-3.07)	0.87
Reported knocking on neighbours' doors	2 (14.3)	6 (15.8)	0.66 (0.14-3.12)	0.60

Reported having visitors	2 (14.3)	9 (23.7)	0.52 (0.10-2.77)	0.44
Reported entering other residents' apartments	2 (14.3)	5 (13.2)	1.10 (0.19-6.44)	0.92
Health care service provided inside apartment	1 (7.14)	3 (7.89)	0.90 (0.09-9.42)	0.93
Food delivery service provided inside apartment	2 (14.3)	3 (7.89)	1.89 (0.28-12.7)	0.51

[a]Odds ratio (OR), Confidence interval (CI)

References

1. **Katoh K, Standley DM**. (2013) MAFFT Multiple sequence alignment software version 7: improvements in performance and usability. *Molecular Biology and Evolution*; **30**: 772–780.

2. **Minh BQ, *et al.*** (2020) IQ-TREE 2: new models and efficient methods for phylogenetic inference in the genomic era. *Molecular Biology and Evolution*; **37**: 1530–1534.

3. **Yu G, *et al.*** (2017) ggtree: an r package for visualization and annotation of phylogenetic trees with their covariates and other associated data. *Methods in Ecology and Evolution*; **8**: 28–36.

4. **Rambaut A, *et al.*** (2020) A dynamic nomenclature proposal for SARS-CoV-2 lineages to assist genomic epidemiology. *Nature Microbiology*; **5**: 1403–1407.
